# Prevalence of HCV, HBV, and HIV Seropositivity among Cadavers Referred to Autopsy Hall of Legal Medicine Bureau of Tehran, Iran

**DOI:** 10.1155/2017/2043840

**Published:** 2017-11-29

**Authors:** Jaber Gharehdaghi, Mohammad Hassan Abedi Khorasgani, Mohammad Hassan Ghadiani, Amir Mohammad Kazemifar, Hassan Solhi, Sadra Solhi

**Affiliations:** ^1^Legal Medicine Research Center, Legal Medicine Organization, Tehran, Iran; ^2^Shahid Beheshti University of Medical Sciences, Tehran, Iran; ^3^Qazvin University of Medical Sciences, Qazvin, Iran; ^4^Arak University of Medical Sciences, Arak, Iran

## Abstract

A large number of dead bodies are referred to forensic autopsy halls for medicolegal examination. They can be a source of transmission of infectious diseases through direct contact or autopsy tools. The main aim of this study was to estimate the virus infection rates in the dead bodies. One thousand consecutive dead bodies that had been referred to autopsy hall of Legal Medicine Bureau of Tehran, Iran, during 2016, were included. The blood samples were analyzed in the laboratory for detection of HBs Ag, HBs Ab, HIV Ab, and HCV Ab, after providing informed consent from legal next of kin of the dead bodies. The general characteristics of the dead bodies were also collected by a checklist. Forty-seven cases of HIV seropositivity, 80 cases of HBs Ag seropositivity, and 97 cases for HCV Ab seropositivity were found. Among them, 27 cases of HIV, 40 cases of anti-HBC positive, and 94 cases of RIBA testing positive for HCV were proved through confirmatory tests. In other words, 2.6% of the dead bodies were infected with HIV, 3.8% with HBV, and 9% with HCV. The total infection rate was 15.5%. This is a worrying risk for pathologist and autopsy technicians.

## 1. Introduction

The autopsy room has always been a potential source of infection. The autopsy surgeons, forensic pathologists, and other persons engaged directly or indirectly in conducting postmortem examination are at greater risk of exposure to blood-borne viruses like human immunodeficiency virus (HIV), hepatitis B virus (HBV), hepatitis C virus (HCV), herpes, hanta virus pulmonary syndrome, small pox, and human T-cell lymphotropic virus type 1, as well as bacterial infections such as tuberculosis and infection from other pathogenic organisms [1]. Many studies have confirmed that, with the cessation of life, certain pathogenic bacteria are released, which if left unchecked may prove hazardous to the personnel [1].

Occupationally acquired infection can have a devastating impact on the health care worker [2]. Knowledge of the risks of infection is therefore essential. Accidental exposures to high risk pathogens are not infrequent. A proper clinical practice may prevent occasional infections [3]. One of the major issues raised in various countries, especially the developing countries, is the problem of the prevalence and spread of infectious diseases, including AIDS and hepatitis B and hepatitis C. Contaminated blood transfusion, the use of contaminated needles, or contact with blood of an infected person can transmit the diseases [4].

On the other hand, doubts must remain as to whether there is a large unrecognized pool of undiagnosed individuals in the general population. Medicolegal necropsies can be an appropriate group for study of the infection rates. Although they are a preselected group, it has been suggested that they act as a surrogate for the general population and may be particularly sensitive to early changes in epidemiology [5]. The mortuary can be a dangerous place for the individual who is ignorant of, or who ignores, the potential hazards at necropsy. He/she put in danger himself/herself, colleagues working in the mortuary, pathologists, and anatomical pathology technicians (who have the highest rate of necropsy related morbidity), visitors to the mortuary (clinical staff and students), and those involved in handling the body (relatives, funeral directors, embalmers, and crematoria staff), or material derived from it (laboratory workers) after necropsy [6]. The decline in mortuary acquired infections such as tuberculosis and blood-borne hepatitis in the past 25 years can be largely attributed to the increased awareness and adoption of safe working practices [6].

The actual prevalence of these diseases among Iranian cadavers remains to be determined. The present study was conducted to assess the prevalence among cadavers that were referred to autopsy hall of the capital city Tehran, Iran, to determine their cause of death.

## 2. Materials and Methods

The current cross-sectional study was conducted in the autopsy hall of Legal Medicine Bureau of Tehran, Iran, during 2016. Total of 1125 cadavers were included in the study. The studied cadavers were all samples which met inclusion criteria of the study. The inclusion criteria were informed consent of legal next of kin of the dead bodies for participation in the study, need to perform autopsy under authorization of examining magistrate, and negative history of any major hepatic diseases.

In our country, medicolegal autopsy is performed under authorization of examining magistrate usually the next day after death by forensic pathologist of Legal Medicine Organization.

Thirty milliliters venous blood was taken from femoral vein of the cadavers after obtaining informed consent from legal next of kin of the dead bodies. The blood samples were analyzed in the laboratory for detection of HBs Ag, HBs Ab, HIV Ab, and HCV Ab. The hemolyzed blood samples (86 samples) were excluded from the study.

HBs Ab was analyzed using ELISA test (Abnova Co., Taiwan). HCV Ab was measured using ELISA test (Diagnostic Automation/Cortez Diagnostics, Inc., USA). HIV Ab was detected using enzyme immunosorbent (EIA) assay (Sigma-Aldrich Co., Germany). To confirm the diagnosis, Western blot and recombinant immunoblot assay (RIBA) tests were performed on positive samples for HIV Ab and HCV Ab, respectively. All the tests were performed in accordance with the manufacturer's instructions and the standard methods described in earlier studies [6]. The general characteristics of the dead bodies were also collected by a checklist.

The infection rate was calculated as the number of persons with positive test divided by total number of the examined bodies *∗* 100.

The resultant data were analyzed using SPSS software via appropriate statistical tests including chi-square test and Fisher's exact test.

## 3. Results 

The collected data of 1039 dead bodies were included in the study. About 80% of bodies were male. The most frequent age group was 20–30 years and older than 60, respectively (Table 1). The frequency distribution of the time passed since death in the studied subjects is shown in Table 2. Most autopsies were performed within 12–24 hours after death. The probable cause of death of the subjects is shown in Table 3.

About 23% of the studied cases were single, and 61.8% were married. The majority of the studied subjects (41.3%) have education lower than primary school and 33.1% have high school education degree. Regarding their occupational status, 31.4% were self-employed, 118 (11.4%) were simple worker, and 43 (4.1%) were unemployed.

There was positive history of hepatitis or AIDS only in 6 cases. Three hundred twenty-eight subjects (31.6%) had positive history of cigarette smoking. Also, 188 subjects (18.1%) had positive report about drug abuse. In 70 subjects (7.6%), history of imprisonment was positive.

Sign of nonmedical injection mark was seen in 90 subjects (8.7%) during postmortem examination. Also, 117 subjects (12.7%) have tattoos in any part of his/her body.

In 67.3% of the subjects, the liver had normal size and weight during autopsy according to the normal measures reported in the texts. It had more than normal size in 17.2% and less than normal size in 2.1% of the studied subjects. The liver seemed cirrhotic in 5.4% of subjects and fatty in 5.2% of them.

One hundred sixty-one samples (15.49%) that were taken from the studied subjects were positive for at least one of the infections (HBV, HCV, and HIV) including 94 cases (9.05%) positive for HCV, 40 cases (3.84%) positive for HBV, 27 cases (2.60%) positive for HIV, 22 cases positive for HIV and HCV, 4 cases positive for HBV and HCV, 2 cases positive for all three tests, and one case positive for HIV and HBV.

About 18% of the studied subjects were female. Among them, a case of HCV positive and 6 cases of HBV positive were found. There was no HIV positivity in them. The prevalence of HCV seropositivity was 20.4-fold higher in men compared to women. The male/female ratio was 1.18 for HBV.

Statistical analysis of the resultant data with chi-square test and Fisher's exact test showed that there is a significant correlation between marital status and HCV seropositivity (*P* value < 0.01). The contamination rate was higher in singles. The same correlation was also found for HIV seropositivity (*P* value < 0.02). The rate was higher in singles, too. There was a significant relationship between the HIV and hepatitis C seropositivity and history of imprisonment (*P* value < 0.01). Also, there was significant relationship between presence of injection mark during postmortem examination with HIV and hepatitis C seropositivity (*P* value < 0.01). There was significant relationship between history of drug abuse with HIV and hepatitis C seropositivity (*P* value < 0.01). There was also significant correlation between body tattooing and HIV and hepatitis C seropositivity (*P* value < 0.01). There was no relationship between the size and appearance of the liver with seroprevalence of HIV, hepatitis B, and hepatitis C.

## 4. Discussion

The necropsy is a valuable investigation in patients who have died from AIDS because it permits clinicopathological follow-up, elucidation of the descriptive clinical pathology and epidemiology of HIV disease, validation of endpoints in clinical trials, assessment of drug efficacy and toxicity, accumulation of tissue for further research, and medical education [6, 7]. HIV serophobia has been documented among staff working in mortuaries handling high risk cases since the 1980s. Although there is no evidence that HIV is readily acquired in the mortuary. Consequently, it is difficult to justify refusal to undertake necropsies on patients with such infections [6]. The risk of seroconversion after occupational exposure will depend upon the viral load within the patient, the volume of fluid inoculated/ingested, and the susceptibility of the health care worker (including whether or not they receive postexposure prophylaxis with zidovudine) [6].

HBV is highly infectious, and transmission can occur following exposure to extremely small volumes of infected blood. However, the risk of occupational acquisition of HBV is extremely low, largely as a consequence of routine preexposure vaccination among health workers.

In contrast, HCV is probably less infectious than HBV, but no vaccine exists. Occupational acquisition of HCV has been reported in health care workers and the rate of transmission after percutaneous exposure is 2.7–10.79 percent [6]. The infection poses a risk for forensic experts and postmortem room workers [8, 9].

The prevalence of HIV and HCV positivity clearly varies with cause of death; the drug overdose group had far more positive results (81 of 107, or 76% of cases) than other categories, for example, car accidents and any other kind of accidents, where only 13 of 109 cases (13%) were positive tests. This is not unexpected and presumably reflects the known association between high risk behavior (e.g., drug addiction or prostitution) and both HIV and HCV infection. People in groups with a high risk of infection are far more likely to die at a younger age and in circumstances which require a medicolegal inquiry [5].

The main aim of this study was to estimate the prevalence of HIV, HBV, and HCV in the bodies referred to Tehran Forensic Dissection Hall. Results of the present study showed that the overall contamination rate with the above-mentioned viruses is 15.49%. The calculated rates for the infections are lower than the studies of Cattaneo et al. [6, 8, 10]. The later study has been performed in Iran among autopsy samples. A study performed on autopsy samples in Jordan has reported the HBV and HCV infection rate of 2.5% which are even lower than the present study [11]. In the present study, the infection with the viruses was correlated with history of drug abuse, imprisonment, male gender, presence of injection mark, and tattooing. However, the contamination is not limited to subjects with these risk factors. So, one cannot rely on past medical history solely to assess the risk factor of the infection. All dead bodies should be considered potentially contaminated and appropriate preventive and protective methods were obeyed during autopsy [12–15].

## Figures and Tables

**Table 1:** Frequency distribution of age groups in the studied cases.

Age groups	Frequency	Percentage	Overall percentage
Less than 20	59	5.7	5.7
20–30	233	22.4	28.1
31–40	166	16	44.1
41–50	152	14.6	58.7
51–60	149	14.3	73.1
More than 60	258	24.9	97.9
unknown	22	2.1	100

**Table 2:** Frequency distribution of the time passed since death in the studied subjects.

Time passed after death (hours)	Frequency	Percentage	Overall percentage
Less than 6	18	1.7	1.7
6–12	173	16.7	18.4
12–24	581	55.9	74.3
24–48	191	18.4	92.7
More than 48	62	6	98.7
unknown	14	1.3	100

**Table 3:** Frequency distribution of the cause of death in the studied subjects.

Cause of death	Frequency	Percentage	Overall percentage
Heart disease	277	26.7	26.7
Trauma	319	30.7	57.4
Internal disease	118	11.4	68.7
Poisoning	64	6.2	74.9
Drug intoxication	81	7.8	82.7
Infectious diseases	34	3.3	85.9
Burn	29	2.8	88.7
Respiratory failure and asphyxia	64	6.2	94.9
Other	53	5.1	100

## References

[1] Yadav A., Pathak D., Alam F., Vyas N. (2014). Seroprevalence of HIV, HBV and HCV among the cadaver population - A Jaipur based study. *Medico Legal Update*.

[2] Burton J. L. (2003). Health and safety at necropsy. *Journal of Clinical Pathology*.

[3] Kiani M., Barzegar A., Bazmi S. (2005). autopsy infections and ways to prevent its transmission. *Journal of Legal Medicine*.

[4] Alavian M. (2003). Investigate the factors associated with hepatitis B vaccination in Tehran physicians. *Journal of Medical Systems*.

[5] Rasoolinejad Y. (2002). Infection in injection drug users. *Journal of Medical Systems*.

[6] Cattaneo C., Nuttall P. A., Molendini L. O., Pellegrinelli M., Grandi M., Sokol R. J. (1999). Prevalence of HIV and hepatitis C markers among a cadaver population in Milan. *Journal of Clinical Pathology*.

[7] Saleh M., Kazemifar A. M., Saleh A. E., Nobari A. H., Samimi R. A. (2011). Prevalence of HIV, hepatitis B and C seropositivity in expired iv drug abusers in Hamadan. *Scientific Journal of Forensic Medicine*.

[8] Khambatta S., Sinha N., Sabale P., Shastri J., Mohite S., Ambiye M. V. (2014). Seroprevalence of HBV and HCV in forensic autopsy cases. *Medico Legal Update*.

[9] Bansal M. K., Naik S. K., Gupta P., Rani Y., Sherwal B. L. (2014). Post mortem and the risk of HIV infection. *Indian Journal of Forensic Medicine and Toxicology*.

[10] Eriksen M. B., Jakobsen M. A., Kringsholm B. (2009). Postmortem detection of hepatitis B, C, and human immunodeficiency virus genomes in blood samples from drug-related deaths in Denmark. *Journal of Forensic Sciences*.

[11] Bakri F. G., Al-Abdallat I. M., Ababneh N., Al Ali R., Idhair A. K., Mahafzah A. (2016). Prevalence of blood-borne viral infections among autopsy cases in Jordan. *Qatar Medical Journal*.

[12] Delmonico F. L. (2000). Cadaver donor screening for infectious agents in solid organ transplantation. *Clinical Infectious Diseases*.

[13] Kadam S. S., Akhade S., Desouza K. (2015). Autopsy practice, potential sources of occupational hazards: A review for safety and prevention. *Journal of Indian Academy of Forensic Medicine*.

[14] Bhullar D. S. (2012). Safety measures in dealing with dead. *Journal of Punjab Academy of Forensic Medicine and Toxicology*.

[15] Hostiuc S., Curca G. C., Ceausu M., Rusu M. C., Niculescu E., Dermengiu D. (2011). Infectious risks in autopsy practice. *Romanian Journal of Legal Medicine*.

